# Heavy Metal Accumulation in Soil Amended with Roadside Pond Sediment and Uptake by Winter Wheat (*Triticum aestivum* L. cv. PBW 343)

**DOI:** 10.1100/tsw.2010.220

**Published:** 2010-12-14

**Authors:** Tanmoy Karak, Pradip Bhattacharyya

**Affiliations:** ^1^ Pollution Control Board, Guwahati-21, Assam, India; ^2^ Production Division, Tocklai Experimental Station, Tea Research Association, Jorhat-8, Assam, India; ^3^ Department of Renewable Resource, University of Wyoming, Laramie, WY, USA

**Keywords:** Triticum aestivum L., heavy metals, soil pollution, sequential extraction, risk assessment code, health risk index

## Abstract

The risks of heavy metal accumulation and the dynamics related to roadside pond sediment application in comparison to control of winter wheat (*Triticum aestivum* L.) were investigated in field experiments. Selective sequential extraction procedures revealed that application of pond sediment in soil increases the labile pools of the studied heavy metals (Cd, Cr, Cu, Ni, Pb, and Zn). Risk assessment codes concluded that Cu and Pb were in the high-risk zone in both pond sediment and soil amended with pond sediment, whereas Zn and Cu were found in the medium-risk zone for control soil. Heavy metal accumulation by wheat straw and grain (39.38, 1.18, 23.73, 0.36, 0.18, and 16.8 mg kg^-1^ for Zn, Cd, Cu, Cr, Ni, and Pb, respectively, for wheat grain) was significantly increased through application of pond sediment. However, metal accumulation did not thwart the enhancement of wheat yield when pond sediment was applied. Health risk indexes of analyzed heavy metals were found to be within the Indian permissible limit for foodstuffs. Pond sediments help to fortify wheat grain by increasing the concentration of Zn and Cu as a source of micronutrients in the diet. However, a significant increase of Pb in wheat grain through pond sediment could be a health concern for its long-term application. Therefore, pond sediment would be a valuable resource for agriculture as an alternative organic supplement, but long-term use may require the cessation of the excavated sediment as agricultural landfill in order to restrict heavy metal contamination through it.

## INTRODUCTION

An artificial, small water body with a typical landscape is known as pond. Ponds are widely distributed in India, Sri Lanka, China, and in some central European countries[1]. The traditional functions of ponds in India are: to use them for pisciculture (also known as fish farming), to use the water for selective domestic purposes, and to use the water supply for irrigation in dry seasons. After long-term human activities, pond water receives different organic substances (e.g., rice, wheat husk and grain, oil cake, cow dung, food waste, leaf litter, poultry litter, dead fish, livestock manure, domestic wastes, feces, dried blood meal, dead snails, vermicompost, etc.), as these organic substances are known as food supplements for fish[2,3,4]. These substances are also deposited as pond sediments along with different particulate matters by human activities. Application of pond sediments to Indian agricultural land for plant nutrition is a common practice, particularly in the state of West Bengal. Karak[5] reported that pond sediments can be effectively used for rice cultivation as a source of different plant nutritional soil supplements. However, the release of state highway pollutants, especially heavy metals, into the roadside pond via storm water runoff is known to cause detrimental effects to the receiving environment[5,6,7]. Therefore, if roadside pond sediments are used for landfill on the agricultural land, then the detrimental effects may occur in soils.

Furthermore, the mobility and bioavailability of heavy metals in soils and bottom sediments strongly depend on their specific chemical and mineralogical forms and their binding characteristics. This implies that these forms and characteristics should be studied rather than their total concentrations[7,8]. The selective sequential extraction (SSE) procedure is used to separate heavy metals in soils and sediments into operationally defined geochemical fractions, such as exchangeable metals associated with carbonates, metals associated with Mn and Fe oxides, metals associated with organic matter, and residual metals[9,10,11,12]. Therefore, SSE is an ultimate pathway to understand the different fractions of heavy metals in sediments and their availability to plants.

Among the cereal crops, wheat is the second principal crop in India, which was the third largest producer in the world for the year 2005[13]. Application of pond sediment has been shown to be promising for rice cultivation[5], however, until now, no report has been available about the application of pond sediments in wheat cultivation. Even though pond sediments supply plant nutrients, some heavy metals present in roadside pond sediments may go into the soil solution, risking their uptake by the wheat crop. The diverse affects of heavy metals on plant growth are well documented. However, studies are scanty on the dynamics of heavy metals in the soil-wheat plant systems by using roadside pond sediment under field conditions that assess wheat plant uptake of heavy metals and the health risks after consumption.

Therefore, the aim of this study was to ascertain the forms of heavy metals in roadside pond sediment and how the forms are linked to the wheat yield, the heavy metal uptake by wheat plants, and the health risks after consumption. This was envisaged to throw light on heavy metal contamination in the soil-wheat plant food-chain system after the application of roadside pond sediment.

## MATERIALS AND METHODS

### Site Description

The manmade pond from which the pond sediments were taken for the present experiment is located at Jalsara (22°40'N, 87°46'E) in the West Medinipur district of West Bengal. The pond is very shallow (maximum depth of the pond is between 0.2 and 1.5 m). Certain features of this pond, such as the steep slopes of the banks, indicate that it may have been altered by human activities; however, the history of this pond was not well documented. The total area of this pond is around 1.25 ha. This pond is situated at the roadside (state highway-4; SH-4) (Fig. 1). Traffic congestion is common on this road as it connects the locations among Mechogram and different parts of the district. The daily traffic density on this road is >1250 vehicles.

This pond is usually used for irrigation purposes (especially in the dry season), domestic purposes, and fishery. Water from this pond receives road dust throughout the year and road runoff during the rainy season. Because land sliding from the bank and roadside of this pond is common, it needs to be excavated every 5 years. Excavated sediment of this pond is shifted to the nearby lowland agricultural field as landfill. This practice is done for land filling by pond sediments and, at the same time, for soil health improvement. In Fig. 1, field A is amended with pond sediments and field B is a control field.

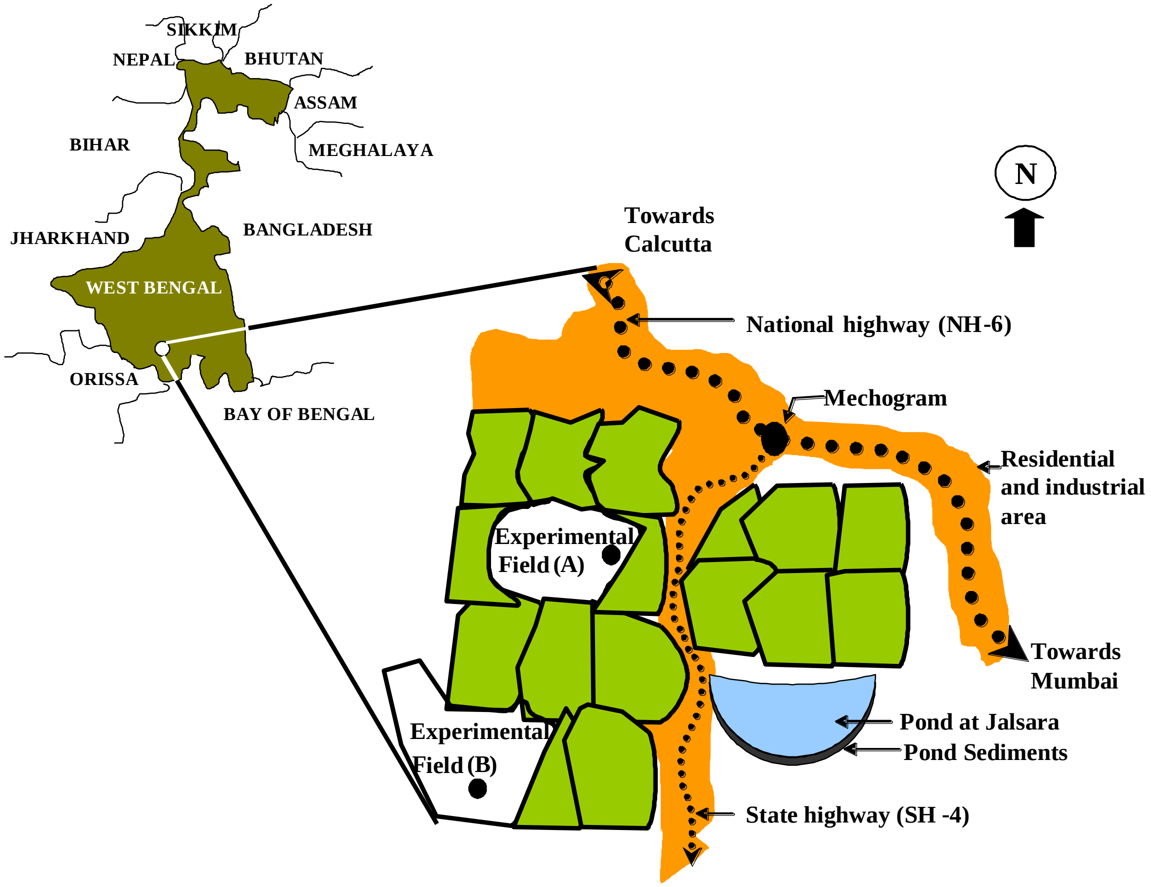

**FIGURE 1.** Schematic location of experiment sites (not in scale).

## Sample Collection, Preparation, and Analysis

The 39 representative sediment samples from the pond were collected randomly using a polyethylene corer, which removed approximately 40 cm of surface sediment. Sediment subsamples were prepared from the 39 representative samples. Sediment subsamples were dried at 105°C overnight, homogenized, screened through a 1.0-mm sieve, and subjected to further analysis.

Field experiments were conducted during the winter of 2006–2007 (December 2006 to February 2007) on wheat (*Triticum aestivum* L., cv. PBW 343) on private agricultural land (field A) by applying the above-mentioned pond sediments at Jalsara, West Medinipur, West Bengal. The site of the experimental soil represented Gangetic alluvial soil (Typic Fluvaquent). Wheat crops were visually monitored throughout the growing season to assess the signs of deficiency or toxicity. A parallel experiment was also performed on an agricultural field (field B, near to field A) without the application of the pond sediments. Therefore, field B was treated as the control treatment field. About 28 cm of pond sediment was used to fill field A; about 28 cm of soil from the upper layer of this field was taken to prepare house and bricks in the summer of 2006. After drying this sediment, field A was subjected to tillage in order to mix the pond sediment with the soil. Tillage was also done in the control plot. Prior to wheat cultivation, rice (*Oryza sativa* L. cv IET-4094) was cultivated and harvested from the field. The rice crop was cultivated just after the land filling, as land filling by pond sediments is usually done in the summer season and, thereafter, the first cultivated crop season was rice. The experimental result on rice cultivation has already been published by Karak[5].

In the field experiments, basal doses of N (105 kg $ha^{-1}$), P (32 kg $ha^{-1}$ as $P_2O_5$), and K (18 kg $ha^{-1}$ as $K_2O$) were also applied to supply N, P, and K, respectively[14]. Wheat plants were harvested when physiologically mature. After harvesting, the plant parts (straw and grain) were collected separately from fields A and B. Straw and grain samples were weighed, then washed with distilled water to eliminate airborne particulate matter. Straw and grain samples were dried in an oven at 65 ± 0.3°C for 24 h and then reweighed to determine water content. Then the samples were ground to powder and used for metal analysis.

Selected chemical characteristics, such as pH, electrical conductivity, percent of clay, cation exchange capacity (CEC, cmol $kg^{-1}$), total organic carbon (g $kg^{-1}$), total nitrogen (g $kg^{-1}$), humas-C (g $kg^{-1}$), and total S (g $kg^{-1}$), in pond sediment/soil from fields A and B were analyzed following the procedure described by Jackson[15] and Nelson and Sommers[16]. To investigate the metal bioavailability in the soil and sediment, the diethylenetriaminepentaacetic acid (DTPA) test for extractable heavy metals (like Pb, Cu, Zn, Ni, Cd, and Cr) was performed as described by Lindsay and Norvell[17]. DTPA was selected for plant-available heavy metals because DTPA is a multidentate chelating agent that has been widely applied for plant-available heavy metals. Furthermore, DTPA forms water-soluble complexes with many heavy metals with high stability constants and is highly significant for plant-available heavy metals[18].

Pond sediment, soil, and plant samples (1 g) were digested for total concentrations of selected heavy metals after adding 15 mL of a triacid mixture ($HNO_3$, $H_2SO_4$, and $HClO_4$ in 5:1:1 ratio) at 80°C until a transparent solution was obtained[19]. Six-step SSE of heavy metals in sediment was adopted from Tessier et al.[10] and is described in Table 1. All the filtrates collected were acidified with $HNO_3$ prior to the analysis with atomic absorption spectrophotometry (AAS). The VARIAN atomic absorption spectrophotometer (Model No.: AA 240, Australia) was used to assess the heavy metals in extracts. The intramethod reproducibility of the SSE procedure was found to be good, with relative standard deviations (RSDs) of <3% for all fractions. These results are very similar to those recorded in the literature for the same extraction scheme[20,21,22,23].

The accuracy and precision of the analyses for heavy metals were checked against the SRM-2709 (San Joaquin soil from the National Institute of Standards and Technology, Gaithersburg, MD), SRM-1645 (river sediment from the National Bureau of Standards, Washington, D.C.), and SRM-1515 (apple leaves, National Institute of Standards and Technology, Japan). Analysis of these SRMs showed that all the certified and reference elements were within the expected values (95–110% recovery). Therefore, the results of the SRMs showed a good agreement with the certified and reference values for all the analyzed elements.

For all the experimental samples, two of the three replicates were analyzed. If the data of two replicates were not within an acceptable range of precision (relative error <5% for high concentrations and <15% for low concentrations), the third sample was analyzed. This was necessary when the analyte was near to detection limit of the analytical procedure.

The health risk index (HRI) was calculated as the ratio of estimated exposure of test crops and oral reference dose[24]. Oral reference doses were 0.3, $1 \times 10^{-1}$, and $4 \times 10^{-2}$ mg $kg^{-1}$ $day^{-1}$ for Zn, Cd, and Cu, respectively[25], and 1.5, 0.02, and 0.004 mg $kg^{-1}$ $day^{-1}$ for Cr, Ni, and Pb, respectively[26]. Estimated exposure is obtained by dividing daily intake of heavy metals by their safe limits.

Daily intake was calculated by the following equation:

$$\text{Daily intake of metal (DIM)} = \frac{C_{metal} \times D_{foodintake}}{B_{averageweight}}$$

where $C_{metal}$, $D_{foodintake}$, and $B_{averageweight}$ represent the heavy metal concentrations in wheat grain (mg $kg^{-1}$), daily intake of wheat grain, and average body weight, respectively. The average daily wheat intake rate of 190 g and average body weight of 60 kg were taken for calculation.

**TABLE 1**
**Multistep SSE Scheme for Determination of Heavy Metal Fractionation in Soil and Pond Sediment Samples**

| Step | Presumed Extracted Phase (Fraction) | Extractant and Experimental Condition |
|---|---|---|
| 1 | Water soluble (F1) | 40 mL $H_2O$, 30-min agitation |
| 2 | Exchangeable (F2) | 40 mL 1 M $MgCl_2$, pH 7, 2-h agitation at room temperature |
| 3 | Acid soluble/carbonate fraction (F3) | 40 mL 1.0 M $CH_3COONa$ (pH 5) and agitate at 20$^oC$ for 5 h |
| 4 | Reducible/ Fe and Mn fraction (F4) | 40 mL 0.04 M $NH_2OH{\cdot}HCl$ in 25% $CH_3COOH$ and agitate at 96$^oC$ for 6 h |
| 5 | Oxidizable/organic matter and sulfide fraction (F5) | 25 mL 30% $H_2O_2$ (pH 2) and 15 mL 0.02 M $HNO_3$ before heat at 85$^oC$ for 2 h followed by 10 mL 3.2 M $CH_3COONH_4$ in 20% $HNO_3$ before diluting to 50 mL and agitate for 30 min |
| 6 | Residual/mineral matrix phase (F6) | 1:1 $HNO_3$, 65% $HNO_3$, 30% $H_2O_2$, and HCl and 95 ± 5$^oC$ |

The IRRISTAT statistical package (version 3/93) developed by IRRI, Philippines was used for statistical analysis. Pearson's correlation coefficient (r) was used to determine the relationship between different fractions of heavy metals in amended and nonamended soil with heavy metal content of wheat straw and grain. Correlation coefficients followed by one and two asterisks are significant at 5 or 1% level, respectively ($p < 0.05$ or $p < 0.01$).

## RESULTS AND DISCUSSION

### Physical and Chemical Properties of Sediments and Soils

Selected physical and chemical properties of sediments and soils are presented in Table 2. The results corroborated that soil from field A before excavation and soil from field B did not show any significant changes in physical-chemical properties. However, the influence of pond sediment application in field A was clearly observed in all the chemical parameters. Both pond sediment and soil were found to be neutral in reaction. The pond sediment was dark brown (10YR 3/3) in color with a rotten egg smell, whereas the soil was found to be slightly gray in color with an earthen smell. Physically, the sediment samples were paste-like with high water-holding capacity (about 62%) and high clay content (72%). Clay content in sediment, pond sediment–amended soil, and in soil (only) was found to be between 42 and 72%. All the samples were clayey, which reflected their identical indigenous source. The high water-holding capacity of the sediment was probably due to the presence of humified carbon (44 g $kg^{-1}$) and clay content. Between the two agricultural fields, the percent of organic carbon was found to be high in soil from field A due to the application of pond sediment. Total nitrogen content in pond sediment was found to be 0.14%, which was significantly higher than agricultural soils. C/N and CEC of sediment were found to be 11.49 and 114 cmol $kg^{-1}$, reflecting the proposed standard of maturity of sediments[27]. The lower C/N ratio indicated that pond sediment was in the acceptable range for soil nutrient amendment[28]. The lower C/N ratio in pond sediments was maybe due to its long period of submergence, which creates a methanogenic phase, and carbon losses were presumably greater than nitrogen losses. The CEC of the sediment exceeded the limit value of 60 cmol $kg^{-1}$ on ash-free basis for mature composts[29] and CEC value was found to be similar to those for tilapia ponds in Thailand[30]. This was expected, since carboxyl groups that contribute greatly to the CEC are formed during decomposition

**TABLE 2**
**Chemical and Physical Characteristics of Pond Sediment and Soils**

| Parameters | | Pond Sediments ± SE[a] | Soil from Field A Before Excavation ± SE | Soil from Field A after Mixing with Sediment ± SE | Soil from field B ± SE |
|---|---|---|---|---|---|
| pH | | 6.3 ± 0.01 | 6.9 ± 0.01 | 6.4 ± 0.06 | 6.9 ± 0.01 |
| EC (dS $m^{-1}$) | | 3.2 ± 0.02 | 0.29 ± 0.01 | 2.8 ± 0.02 | 0.26 ± 0.01 |
| Sand (%) | | 16 ± 0.06 | 27 ± 0.02 | 20 ± 0.05 | 30 ± 0.02 |
| Silt (%) | | 12 ± 0.09 | 31 ± 0.02 | 14 ± 0.06 | 29 ± 0.01 |
| Clay (%) | | 72 ± 0.29 | 42 ± 0.13 | 66 ± 0.19 | 41 ± 0.14 |
| Organic carbon (g $kg^{-1}$) | | 162 ± 0.98 | 12 ± 0.11 | 158 ± 0.83 | 12 ± 0.12 |
| Total nitrogen (g $kg^{-1}$) | | 14.1 ± 0.13 | 1.6 ± 0.01 | 14.5 ± 0.11 | 1.4 ± 0.01 |
| C/N | | 11.49 ± 0.12 | 7.50 ± 0.05 | 10.90 ± 0.11 | 8.57 ± 0.04 |
| Humus-carbon (g $kg^{-1}$) | | 44 ± 0.39 | — | 38 ± 0.28 | — |
| Total sulfur (g $kg^{-1}$) | | 12.3 ± 0.02 | 0.2 ± 0.01 | 11.3 ± 0.03 | 0.2 ± 0.01 |
| CEC (cmol $kg^{-1}$) | | 114 ± 1.23 | 22 ± 0.12 | 108 ± 1.49 | 21 ± 0.26 |
| Total metals) (mg $kg^{-1}$) | Zn: | 356.0 ± 2.14 | 86 ± 1.15 | 301 ± 2.14 | 82 ± 1.06 |
| | Cd: | 15.0 ± 0.02 | 0.23 ± 0.01 | 5.8 ± 0.04 | 0.21 ± 0.01 |
| | Cu: | 121.0 ± 1.11 | 82 ± 0.98 | 128 ± 0.92 | 89 ± 0.52 |
| | Cr: | 13.0 ± 0.01 | 1.3 ± 0.01 | 11.3 ± 0.01 | 1.6 ± 0.01 |
| | Ni: | 15.0 ± 0.12 | 1.9 ± 0.01 | 12.3 ± 0.23 | 1.4 ± 0.05 |
| | Pb: | 121.0 ± 2.98 | 2.9 ± 0.98 | 110 ± 1.94 | 2.9 ± 0.86 |
| DTPA extractable metals (mg $kg^{-1}$) | Zn: | 113.0 ± 1.26 | 24 ± 0.87 | 110.2 ± 1.11 | 24 ± 0.68 |
| | Cd: | 2.3 ± 0.23 | BDL[b] ± - | 1.9 ± 0.19 | BDL ± - |
| | Cu: | 51.0 ± 0.35 | 19 ± 0.11 | 13.5 ± 0.28 | 21 ± 0.36 |
| | Cr: | 1.3 ± 0.01 | BDL ± - | 0.9 ± 0.01 | BDL ± - |
| | Ni: | 0.9 ± 0.01 | BDL ± - | 0.2 ± 0.01 | BDL ± - |
| | Pb: | 58.0 ± 0.97 | BDL ± - | 52.0 ± 0.29 | BDL ± - |

[a]SE = standard error; [b]BDL= below detectable limit.

of pond sediment in a highly reducing condition. Boyd and Sommers[31] also documented the high CEC in pond sediments, which was related to native characteristic of the soils[32]. The soils from field B reported moderate CEC, indicating average metal-retention capabilities (21 ± 0.26 cmol $kg^{-1}$). Chary et al.[33] reported that moderate CEC of soils ranged between 22.7 and 29.2 cmol $kg^{-1}$ when soil was irrigated with sewage water in the Musi River basin in India. The total sulfur content in pond sediment was 12.3 g $kg^{-1}$. A high amount of sulfur (30.03 mg $kg^{-1}$) was also reported by Thunjai et al.[30] when pond sediment was collected from a tilapia pond in the Samutprakarn Province of Thailand. The high amount of sulfur in sediment was probably due to the application of mustered cake in ponds as an important nutrient for fish. A comparatively lower amount of sulfur was found in agricultural soil. Based on the properties like color, pH, clay content, CEC, and total humus, the sediment could be graded as mature compost[27].

In general, total heavy metals in pond sediment were found to be higher than in agricultural soil (Table 2). The significant amount of heavy metal content in this sediment can be explained by the fact that the sediment was collected from a roadside pond that receives road dust and roadside runoff during the rainy season. Besides this, a high amount of clay content in sediment is also highly responsible for retaining heavy metals in aquatic sediments[34]. The values of heavy metals in soil amended with pond sediment have shown a remarkable increase compared to nonamended soil, indicating the increase in the metal contents contributed by pond sediment application. In the sediments, soils, and soils amended with pond sediment, the range of Zn was between 356.0 ± 2.14 and 82 ± 1.06 mg $kg^{-1}$. Chary et al.[33] reported that a similar trend of Zn contamination (227 to 401 mg $kg^{-1}$) was there in the soil of the Musi River basin in India that received sewage water for irrigation. In this study, a fourfold increase of Zn concentration was observed in amended soil than that of control soils. Zn concentration in all the samples was found to be in the permissible limits of Indian standards, where Zn concentration was prescribed between 300 and 600 mg $kg^{-1}$[35]. Cu followed the trend next to Zn and showed 18–50% of analyzed total heavy metal concentration. A further 47–50% of Cu was contributed in agricultural soils with respect to the total heavy metals. The range of total Cu among the investigated samples was 82–128 mg $kg^{-1}$. The concentration of Cu in pond sediment was 1.5-fold higher than that of the Cu background value in Jalsara soils (Table 2). The concentration of Cu in field B was higher than in the pond sediment, which may be due to the cumulative effect of soil contamination. The recommended limit for total Cu concentration in soils in India is 135–270 mg $kg^{-1}$, based on the ecotoxicological data and, therefore, Cu concentration in sediment and soil was within the safe limit[35]. However, total Cu concentration in sediment exceeds tolerable levels of Cu (100 mg $kg^{-1}$) described by Kabata-Pendias and Pendias[36]. Therefore, pond sediment requires environmental investigation. Bhattacharyya et al.[37] reported that the concentration of Cu in rice soil increased significantly when the field experiment was conducted with municipal solid waste compost derived from Calcutta city wastes.

Application of pond sediments in agricultural soil profoundly increased Pb concentration. Pb contamination in roadside pond sediment can be described by the type of road near the receiving waters and the overall design of the drainage system[22]. As pond sediment in the present experiment was collected from the roadside pond, traffic congestion, vehicle smoke, and runoff leachate coming from the road during the rainy season may accumulate in the pond sediment. About a 6% increase of Pb concentration over background soils was found when soil was treated with pond sediment. Total Pb concentration in pond sediment and soil amended with pond sediment exceeded the tolerable limit of soil (100 mg $kg^{-1}$)[36]. In this concentration range, toxicity effects from Pb should be observed in some sediment-dwelling organisms, especially sensitive species[38]. A similar finding was also documented by Walter and Cuevas[39] when municipal sewage sludge was applied to soil located on the Central Region of Spain. A similar trend of Cr and Ni was found in all the samples. As it was expected, both Cr and Ni concentrations were significantly increased in soil amended with pond sediments. Total heavy metal load in these two soils was also found in the same trend. Therefore, it is clear that source and mode of heavy metal contamination in these two fields were identical in manner.

The common range of heavy metals for agricultural soil described by Lindsay[40] are as follows: Cr, 1–1000 mg $kg^{-1}$; Ni, 5–500 mg $kg^{-1}$; Cd, 0.01–0.70 mg $kg^{-1}$; Pb, 2–200 mg $kg^{-1}$; Cu, 2–100 mg $kg^{-1}$; and Zn, 10–300 mg $kg^{-1}$. The content of heavy metals in the experimental soil (soil from field B) was found within the prescribed range described by Lindsay[40]. However, the sediment contained a significant amount of heavy metals. Therefore, the sediment used in this experiment can be regarded as contaminated sediment.

Among the single extraction methods, DTPA is the most widely used extractant[41,42]. DTPA was used to estimate plant-available heavy metal, as the DTPA extractant is a more popular reagent to extract bioavailable metals and to predict plant uptake[43]. Recent studies have been proposed for routine soil testing and evaluation of plant-available heavy metal pools in soil by using buffered and neutral salts, since DTPA overestimates this fraction[44,45,46]. However, few data are currently available to assess these new approaches and their comparison with DTPA adequately. For example, Burgos et al.[46] did not compare plant-available heavy metals in their study with widely used DTPA. Furthermore, DTPA extractant contains a high concentration of neutral salt, such as $CaCl_2$, of which $Ca^{2+}$ may exchange rapidly with bivalent cation from soils. DTPA contains triethaloamine (TEA), which is protonated at pH 7.3 and could exchange with cations from the exchange sites, as suggested by Lindsay and Norvel[17]. $CaCl_2$ is the primary component of soil background electrolytes. The exchangeable cations and heavy metals may be displaced by the basic cations ($Ca^{2+}$) commonly present in the extraction solution[47]. Therefore, in this present investigation, we used DTPA for assessing plant-available heavy metals in soil. A significant amount of DTPA-extractable Pb, Zn, and Cu was found in sediment. However, the contents of DTPA-extractable Cr, Cd, and Ni were found to be significantly low, indicating the low extractability of Cr, Cd, and Ni in the sediment. Therefore, the continuous application of this sediment in soil may potentially exacerbate the accumulation of Pb, Zn, and Cu in agricultural soils[21]. DTPA-extractable Pb in natural substance is unusual; however, a significant amount of DTPA-extractable Pb was found in the pond sediment and soil amended with pond sediment. It was expected, as the combustion of leaded petrol had been the main source of atmospheric Pb, and the level of atmospheric Pb has a predominant influence on plant Pb concentrations[48]. The short-distance influence of road transport is likely to contribute significantly to the variation of Pb concentrations in pond sediment vis-à-vis the soil amended with pond sediment.

Only Cu and Zn were available in the DTPA-extractable form in soil A and soil B. Even though Cu and Zn are known as heavy metals in the environment, these metals are also known as micronutrients for plant growth. DTPA-extractable Cu was found, 82 mg $kg^{-1}$, in both the soils, which revealed that all acid-digestible Cu is present in the DTPA-extractable form. DTPA-extractable Zn was found, 24 mg $kg^{-1}$. DTPA-extractable Cu and Zn in soils fall within the normal range of soils as reported by Lindsay[40]. The addition of pond sediments to soil A significantly increased the DTPA-extractable Zn, Cu, and Pb as shown in the sediment. The contents of DTPA-extractable Cr, Cd, and Ni in soil A amended with sediment were 1.9, 0.9, and 0.2 mg $kg^{-1}$, respectively. This indicates the low extractability of Cr, Cd, and Ni in sediment and these elements are quite immobile. The DTPA-extractable Cr, Cd, and Ni was much below the maximum allowable limit of cultivated soils[47]. Fuentes et al.[49] reported similar findings while working with sewage sludge–amended soil.

## SSE of Heavy Metal in Sediment and Soil

Fig. 2 shows the SSE patterns for pond sediment compared with the agricultural soil and soil amended with pond sediment. Each fraction is presented as a percentage of all fractions. Metals extracted from the first three fractions (F1, F2, and F3) are referred to as the labile fraction[50] and the last three fractions (F4, F5, and F6) are referred to as the nonlabile fraction[51]. SSE shows that, apart from increasing metal levels, amending the soil with pond sediments also alters the distribution of metals. In the control soil (soil from field B), the studied heavy metals were predominantly present in the residual fraction. The percent contribution of metals in the labile fraction follows the order Pb (48%) > Cu (42%) > Zn (31%) > Cd (16%) > Cr (9%) > Ni (6%) in the pond sediment. A significant amount of Cd (5.1 mg $kg^{-1}$) and Ni (3.9 mg $kg^{-1}$) was found in the sulfide fraction. This can be described as follows: On sediment analysis, it was observed that sediment is wealthy in total sulfur (Table 2) and this sulfur can be reduced to sulfide ion ($S^{2-}$) by sulfate-reducing bacteria in anaerobic wetland conditions[52]. In this situation, Cd and Ni reacting with $S^{2-}$ leads to formation of highly insoluble Cd and Ni sulfides[53].

Indigenous soil collected from field A shows that only 27% Zn of total Zn and 23% Cu of total Cu are present in the labile fraction. Other metals (Cr, Cd, Ni, and Pb) in this soil present in the nonlabile fraction. The percent contribution of metals in the labile fraction follows the order Cu (48%) > Pb (47%) > Zn (36%) > Cd (27%) > Cr (7%) > Ni (2%) in the soil A amended with pond sediment. This trend follows that similar to sediment after only slight exception with Cu and Pb. Soil collected from field B shows only 29 and 25% of Zn and Cu, respectively, in the labile fraction. Similar to soil A, other metals (Cr, Cd, Ni, and Pb) are also present in the nonlabile fraction in soil B. In all cases, the metal concentrations in the residual fraction are higher than any of the preceding extraction.

The dominant proportion (46–47%) of Pb in sediment and soil A after mixing with sediment was found in the labile fraction (i.e., F1, F2, and F3), which indicates that Pb is present in water-soluble exchangeable forms and bound to carbonates. Other studies also reported that Pb in sediments is associated with labile fractions[38,54,55]. On the contrary, Stead-Dexter and Ward[22] reported that Pb in sediments is strongly associated with Fe-Mn fractions. A high amount of nonlabile metals in sediment can be attributed by sediment pH, and is also influenced by a range of sediment constituents, including clay content and Mn, Al, and Fe oxides and hydroxides[56]. Sediment organic matter is usually identified as the sediment constituent on metal retention[57]. Compared to native soil organic matter, sediment-derived

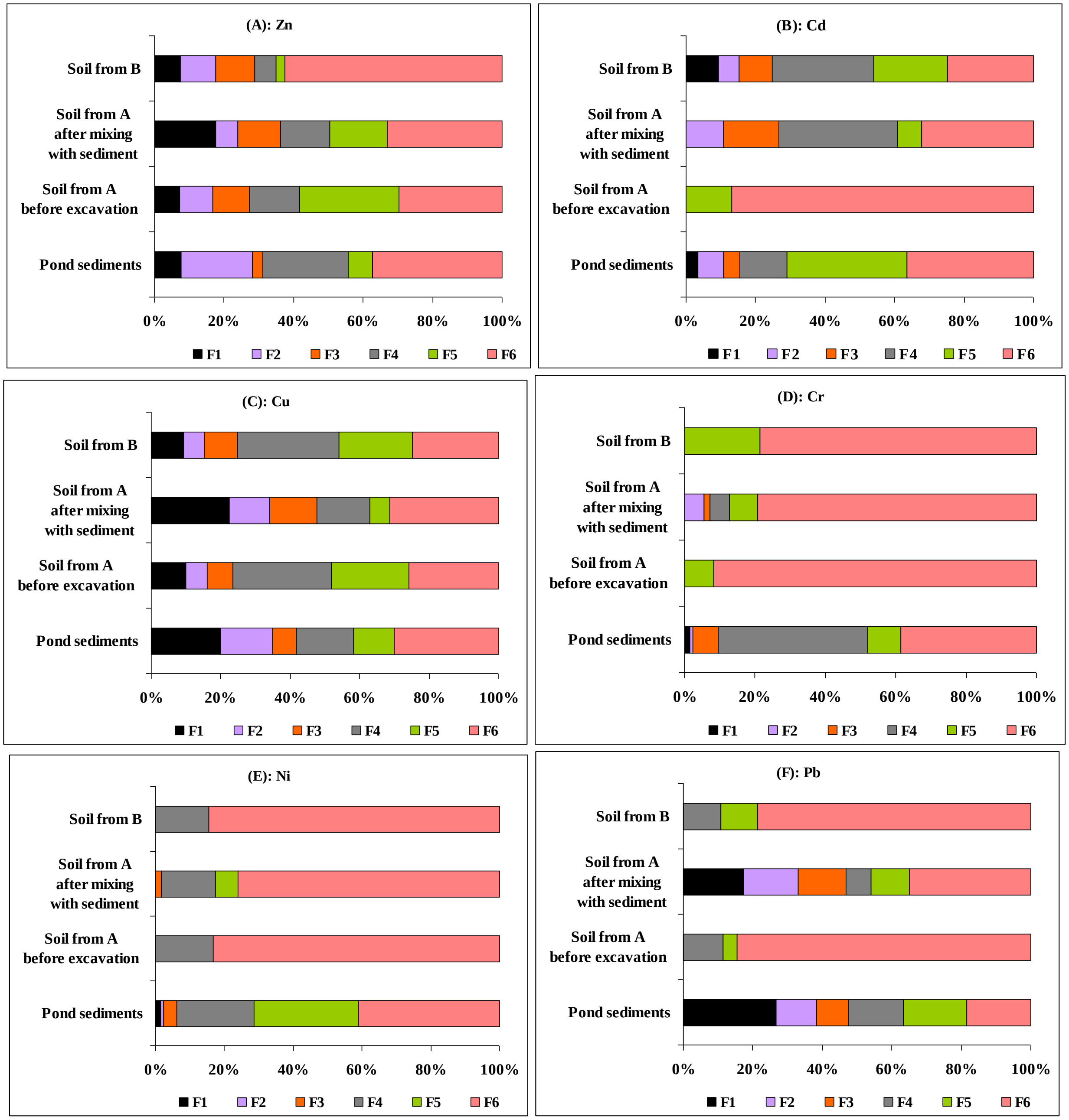

**FIGURE 2.** SSE of discussed heavy metals in investigated soils and sediment.

humic compounds seem to be more recalcitrant[58], and also have smaller contents of carboxyls and phenolic hydroxyls, the functional groups that are important for metal retention[31].

Most of the metals in soil A before excavation and in soil B were distributed in nonlabile fractions (i.e., F4, F5, and F6). Metals confined in the residual fractions are usually not expected to be released over a short period of time under the conditions usually encountered in nature[59]. The nondetectable concentration of Cd, Cr, Ni, and Pb in the exchangeable fraction in soil A and B suggests poor availability of the metals in the soil, which can be attributed to lower acidic to neutral pH of soil. By studying the distribution of the metals between the different phases, their bioavailability and toxicity can be ascertained. The fractions introduced by man's activity include the adsorptive and exchangeable, and those bound to carbonates, which are considered to be weakly bound and may equilibrate with the aqueous phase, thus becoming more rapidly bioavailable[60]. It is evident from the results of the fractionation studies that the metals in the sediments are bound to different fractions with different strengths. The strength values, therefore, give a clear indication of sediment reactivity, which in turn assesses the risk connected with the presence of metals in an aquatic environment[51].

## Ecological Risk Assessment

In order to assess the metal concentrations accumulated in the sediments and agricultural soil from a regulatory perspective, the risk assessment code (RAC) was first adopted by Singh et al.[61]. RAC gives an idea of the possible risk by applying a scale to the percentage of metals presented in water-soluble (F1), exchangeable (F2), and carbonate fractions (F3):

(i.e., $\sum_{n=1}^{3} \mathrm{F}n$ ), i.e., labile phases in sediments. This classification is shown in Table 3.

**TABLE 3**
**Classification of RAC[61]**

| Risk Classification | Metals (%) |
|---|---|
| No risk | <1 |
| Low risk | 1–10 |
| Medium risk | >10–30 |
| High risk | >30–50 |
| Very high risk | >50 |

Following this classification, the risks of exposure to the six heavy metals in this study are depicted in Fig. 3. In pond sediment, Cr and Ni were found in the low-risk, Zn and Cd in the medium-risk, and Cu and Pb are in the high-risk zone. A similar kind of finding was reported by Jain[51] while working on a metal fractionation study of Yamuna River sediments (India) to discuss RAC and found 30–50% of Pb in exchangeable fraction, indicating high risk. Both Zn and Cu fall in the medium-risk zone for soil A and B; however, the other four elements were in the no-risk zone in these soils. A drastic change of RAC was observed when soil A was amended with pond sediment. In this case, Ni was found in the no-risk zone, Cr in the low-risk zone, Cd in the medium-risk zone, and Zn, Cu, and Pb in the high-risk zone.

## Yield of Grain and Straw

The yield parameters (straw and grain) obtained for the two fields are depicted in Fig. 4. The results show that pond sediment helped to boost wheat yields significantly ($R^2 = 0.978^{**}$) over nonamended soil. The percent increase of wheat grain and straw through pond sediment application over control soil was 55.45 and 50.78%, respectively. This significant increase could be ascribed to the greater amount of nutrient added through pond sediment. Tejada and Gonzalez[62] reported that application of compost originating from crushed cotton gin residues increased significant yield of wheat under dryland conditions.

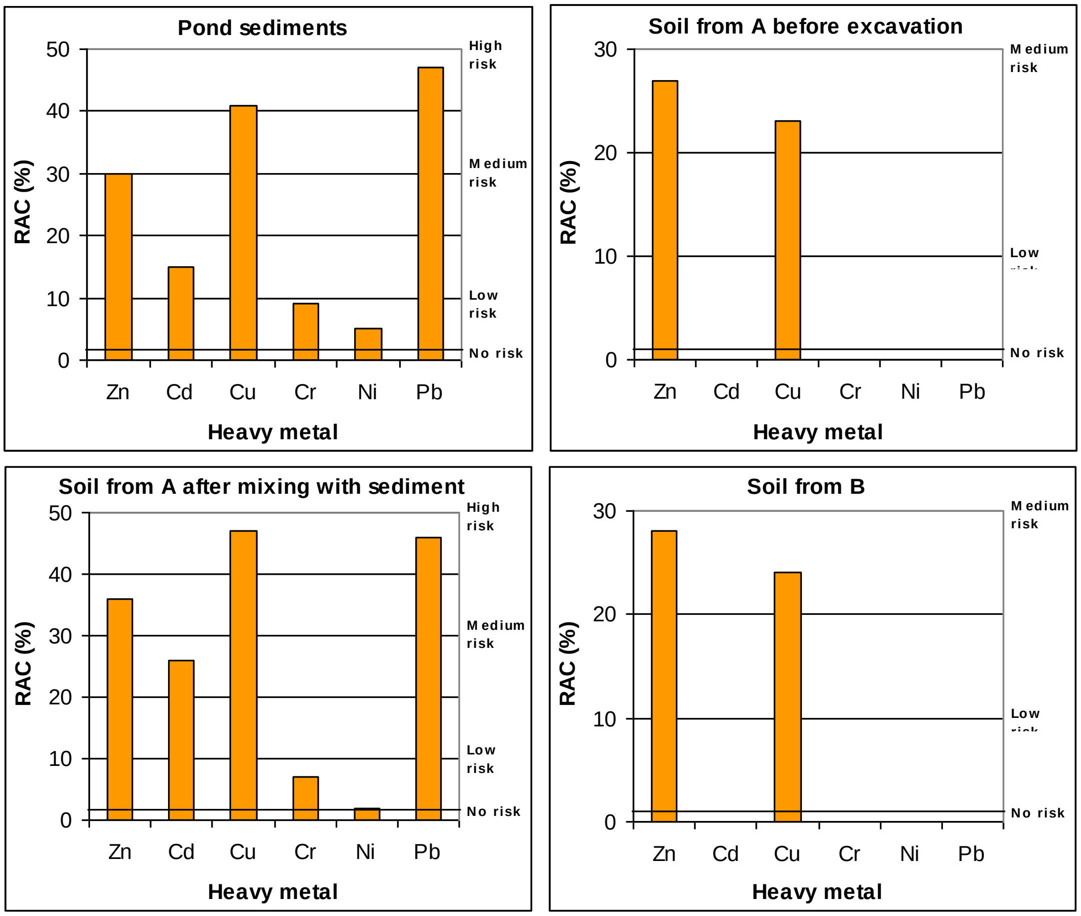

**FIGURE 3.** Percent of RAC in experimental samples.

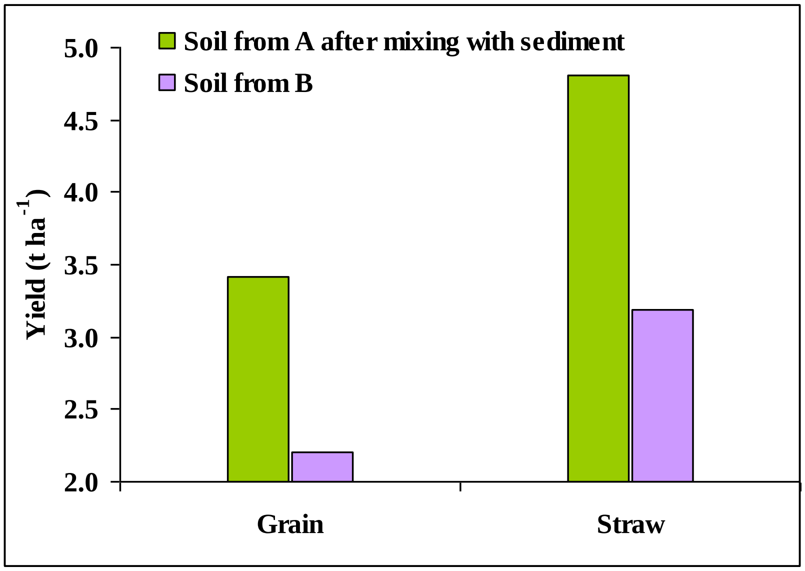

**FIGURE 4.** Wheat grain and straw yield in control soil and soil amended with pond sediment.

## Heavy Metals in Wheat Straw and Grain

The heavy metal concentration in wheat grain and straw is presented in Fig. 5. Heavy metal concentrations in wheat grain and straw were lowest in soil B (i.e., nonamended soil). Straw accumulates a higher concentration of heavy metals than grain. This indicates that heavy metal uptake in wheat is exclusively distributed or translocated to the tissues. Zn, Cu, and Pb were found to be the more dominant metals in wheat grain and straw. Similar findings have been summarized in different research work when wheat plant is grown in sewage sludge–amended irrigated farmland[63,64,65]. Heavy metals in the grain amended with sediment were decreased in the order Zn > Cu > Pb > Cd > Cr > Ni. This result corroborates the RAC values and the labile fraction of heavy metals in sediment. Therefore, the labile fraction of heavy metals in sediment significantly influenced the content of heavy metals in the wheat grown in sediment-amended soil. However, the labile fraction of heavy metals in soil is responsible for phytoavailability, which could not maintain the uptake of heavy metals by wheat plants grown in soil B. In soil B, the results of SSE show that only Zn and Cu are present in the labile pool; however, all the selected heavy metals were taken up by the wheat plant. In the reduced soil environment of the wheat crop, heavy metals present in soil may go into the soil solution, risking their uptake by the wheat crop[36]. Moreover, the decomposition of organic matter in tropical climates is faster, which may result in the more available heavy metals to plants.

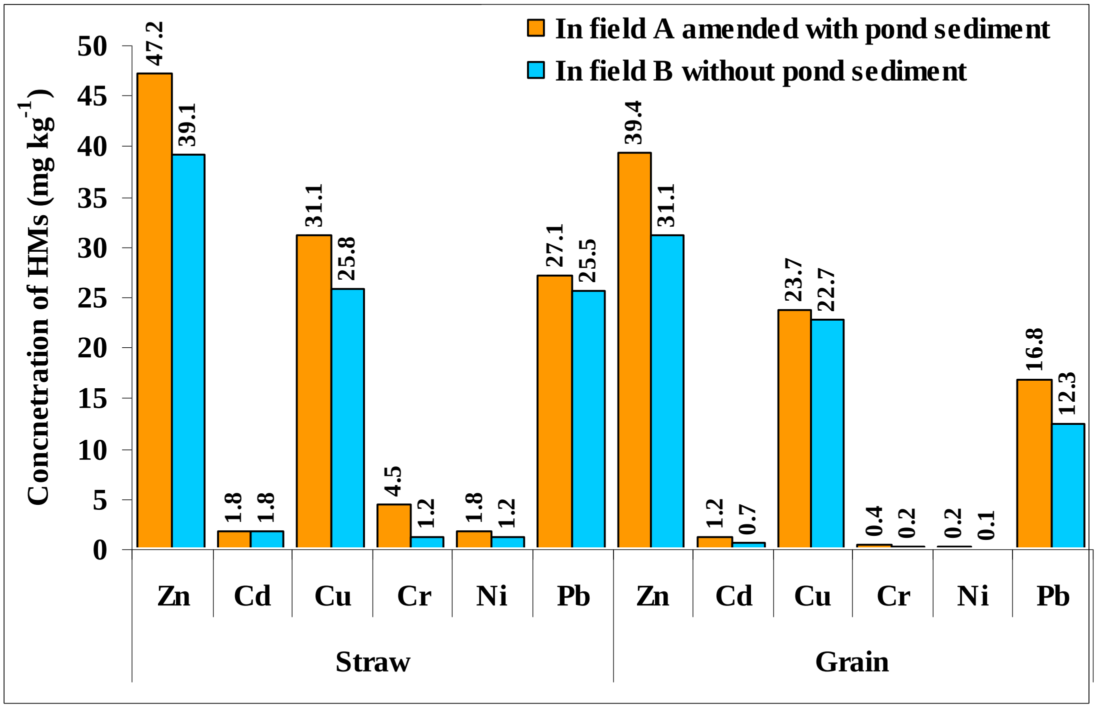

**FIGURE 5.** Heavy metal concentration (mg $kg^{-1}$) in straw and grain of wheat grown under pond sediment–amended and –nonamended soil.

The significant amount of Zn and Cu in wheat grain after application of pond sediment has a special importance in the health perspective, as Zn and Cu both fulfill many biochemical functions in the human metabolism. In a global context, deficiencies of essential trace elements are of main concern[66]. Therefore, application of pond sediment in wheat cultivation may have a beneficial role for the fortification of wheat grain with Zn and Cu through wheat grain consumption. The uptake of heavy metals may be compared with the published threshold values that are considered excessive or toxic for mature plant tissue[36]. Compared with the limiting values of heavy metals, it was observed that the lower concentrations of heavy metals are there in wheat grain and straw. For Zn, the values considered as toxic are listed as 100–400 mg $kg^{-1}$ dry weight, which is higher than the values observed for wheat grain, 39.38 and 31.13 mg $kg^{-1}$, grown in sediment-amended soil and control soil (B), respectively. For Cu, the limiting values are 20–100 mg $kg^{-1}$, for Pb 30–300 mg $kg^{-1}$, for Cr 30 mg $kg^{-1}$, for Cd and Ni 2 mg $kg^{-1}$[36]. Again, wheat grain and straw exhibited values within this range when grown in sediment-amended soil. The different heavy metal fractions were also correlated with the heavy metal concentration in wheat straw and grain. From a statistical analysis, the labile pool of Zn, Cu, and Pb in pond sediment–amended soil was best correlated with straw (r = 0.93*, 0.98**, and 0.89* for Zn, Cu, and Pb, respectively) and grain (r = 0.86*, 0.91**, and 0.84* for Zn, Cu, and Pb, respectively). However in the case of nonamended soil, the labile pool of Zn and Cu was found to be significant in both the wheat straw (r = 0.89* and 0.96** for Zn and Cu, respectively) and grain (r = 0.79* and 0.93** for Zn and Cu, respectively).

## Health Risk Index (HRI)

To assess the comparison of health risk associated with heavy metal contamination of wheat grown in amended and nonamended soils with pond sediment, estimated HRI was calculated (Table 4). The results showed that heavy metal contamination in wheat grown in pond sediment–amended soil had higher HRI in comparison to nonamended soils. However, no heavy metals have shown HRI >1 and, therefore, consumption of wheat grain grown in pond sediment–amended soil has no risk after consumption and is considered as safe for human health[25]. In both fields, Cr showed higher HRI values. A similar kind of finding was reported by Singh et al.[67] of wheat grown by a wastewater-irrigated site at the Dinapur sewage treatment plant situated in a suburban area in the northeast of Varanasi city in the eastern Gangetic plains of India. Cui et al.[24] also reported that local residents of an area near a smelter in Nanning, China had been found to be in no risk due to heavy metals after consumption of wheat.

**TABLE 4**
**Comparison of HRI of Heavy Metals via Intake of Wheat Grain Grown under Pond Sediment–Amended and –Nonamended Soil**

| Wheat Location | Zn | Cd | Cu | Cr | Ni | Pb |
|---|---|---|---|---|---|---|
| Field A (amended with pond sediment) | 3.04E-03 | 1.94E-03 | 5.58E-04 | 2.06 | 1.05E-01 | 1.02E-04 |
| Field B (without pond sediment) | 2.41E-03 | 1.07E-03 | 5.32E-04 | 1.32 | 3.51E-02 | 7.52E-05 |

## CONCLUSIONS

The present experiment was carried out to assess the fate of heavy metals when soil is amended with roadside pond sediment and its relation on heavy metal uptake by winter wheat. Amendment of agricultural lands with roadside pond sediment led to the accumulation of heavy metals in the soil, wheat straw, and grain samples. The chemical forms of heavy metals in sediment and soils were mainly dominated by the reducible, sulfide, and residual fraction. Application of pond sediment increased wheat yield over the control soil. The RAC analysis showed that Cr and Ni were found in the low-risk, Zn and Cd in the medium-risk, and Cu and Pb in the high-risk zone for pond sediment. Use of pond sediments increased the major micronutrients such as Zn and Cu in wheat grain. The HRIs of heavy metals also suggested that heavy metal concentration in grain was highly influenced by pond sediment application. The study suggests that all the analyzed heavy metals in wheat grain were within the Indian permissible limit in foodstuffs. Finally, it is concluded that the application of roadside pond sediment can be used as an organic supplement for resource recovery to soils, which did not necessarily cause short-term problems to the wheat crop. However, further research is needed to confirm the benefits of pond sediment use and optimized dose needed, mainly through long-term control of heavy metal effects on wheat yield and quality.

## ACKNOWLEDGMENTS

The authors acknowledge the financial support of Nature's Beckon, an environmental activist group in North East India. Furthermore, the authors gratefully acknowledge the helpful comments and suggestions made by the two anonymous reviewers of this manuscript. The authors also acknowledge Dr. Sudripta Das, TES, TRA and Dr. Sampa Das, Dibrugarh Polytechnic, Assam for their valuable suggestions.
